# Supplementary material for: Content-rich biological network constructed by mining PubMed abstracts
Source: BMC Bioinformatics. 2004 Oct 8;5:147. doi: 10.1186/1471-2105-5-147 (PMC528731; doi:10.1186/1471-2105-5-147)
Supplement: Additional File 2 — The original results of the above study (non-essential files are deleted to keep the file size under the limit set by BMC bioinformatics). [file 1471-2105-5-147-S2.bz2 › chilibotAdditionalFile2/dip20/574ID7703225E10941/html/NTF3_BDNF.html]

 


 **NTF3** and **BDNF** 
  
Found 1139 abstracts in PubMed, retrieved 20.  
 

 What does Google say? 
 PDF only 
| .edu only 

---

**Interactive relationship** (e.g. stimulation, inhibition, etc)

**Non-interactive relationship** (e.g. studied together, co-existance, homology, etc.)

- Cultured Xenopus spinal cord neurons were treated with a mixture of  **BDNF** , glia derived neurotrophic factor, NT 3  [ **NTF3** ] , and NT 4, in addition to forskolin and IBMX or the cell permeant form of cAMP, to elevate the cAMP level.  Ref: 12832528 J Neurosci, 2003
- LR or DR alter the expression of the mRNAs for NGF,  **BDNF**  and NT3  [ **NTF3** ]  and CNTF within the visual system.  Ref: 12855194 Brain Res Dev Brain Res, 2003
- NGF,  **BDNF** , NT3  [ **NTF3** ] , and NT4 were localized in the lacrimal gland acini with differing cellular distributions.  Ref: 12882781 Invest Ophthalmol Vis Sci, 2003
- Our results suggest that overexpression of  **BDNF** , NT 3  [ **NTF3** ] , trkB and trkC in motoneuron areas neighboring the site of mechanical compression may represent compensatory changes in response to the compromised neuronal function at the level of compression,.  Ref: 12774239 Acta Neuropathol Berl , 2003
- In all studied species both NGF and NT 3  [ **NTF3** ]  like immunoreactivity IR were present in the enteric nervous system, while  **BDNF**  like IR was never detected.  Ref: 12809982 Neurosci Lett, 2003
- We first examined the relative potency of the neurotrophins, brain derived neurotrophic factor  [ **BDNF** ]   **BDNF** , neurotrophin 3  [ **NTF3** ]  and nerve growth factor to prevent glutamate mediated apoptosis.  Ref: 12829403 Neurotox Res, 2002
- We demonstrate that mRNA for brain derived neurotrophic factor  [ **BDNF** ]   **BDNF** , but not neurotrophin 3  [ **NTF3** ]  or nerve growth factor NGF, is readily detectable in PBMC.  Ref: 12753507 Scand J Immunol, 2003
- Here, we examine how pulpal cells and 3T3 fibroblasts overexpressing certain neurotrophic factors nerve growth factor, brain derived neurotrophic factor  [ **BDNF** ] , neurotrophin 3  [ **NTF3** ] , neurotrophin 4, glial cell line derived neurotrophic factor or neurturin influence survival and growth of single trigeminal ganglion neurones in vitro in quantitative terms.  Ref: 12770558 Neuroscience, 2003
- As the VMN undergoes sexual differentiation, we compared  **BDNF** , NGF, NT 3  [ **NTF3** ] , and trkB mRNA expression in the VMN in males and females at embryonic day 20 and PND 4, but found no differences between them.  Ref: 12824057 Brain Res Mol Brain Res, 2003
- To determine which of the neurotrophins NTs nerve growth factor NGF, brain derived neurotrophic factor  [ **BDNF** ]   **BDNF** , neurotrophin 3  [ **NTF3** ]  NT3  [ **NTF3** ] , and neurotrophin 4 5 NT4 and their receptors NTrs, TrkA, TrkB, TrkC, and p75, are present in the adult rat lacrimal gland.  Ref: 12882781 Invest Ophthalmol Vis Sci, 2003
- The expression pattern of neurotrophins changes with age, with falling NGF, NT3  [ **NTF3** ]  and NT4 but increasing granule cell  **BDNF** .  Ref: 12879965 Cerebellum, 2002
- We recently found that human amniotic epithelial HAE cells secrete biologically active neurotrophins such as brain derived neurotrophic factor  [ **BDNF** ]  and neurotrophin 3  [ **NTF3** ] , both of which exhibit trophic activities on dopamine DA neurons.  Ref: 12865158 Brain Res, 2003
- Stored NGF,  **BDNF**  and NT 3  [ **NTF3** ]  was demonstrated by Western blotting and flow cytometry.  Ref: 12752594 Clin Exp Allergy, 2003
- For this purpose, serial immunohistochemical and immunoblot analyses were performed for the expression levels of endogenous brain derived neurotrophic factor  [ **BDNF** ]   **BDNF** , neurotrophin NT 3  [ **NTF3** ] , and their receptors, trkB and trkC in 24 tip toe walking Yoshimura mice twy twy aged 12 24 weeks.  Ref: 12774239 Acta Neuropathol Berl , 2003
- The proteins of the mammalian neurotrophin family nerve growth factor NGF, brain derived neurotrophic factor  [ **BDNF** ]   **BDNF** , neurotrophin 3  [ **NTF3** ]  NT 3  [ **NTF3** ]  and neurotrophin 4 5 NT 4 5 were originally identified as neuronal survival factors.  Ref: 12787574 Prog Neurobiol, 2003
- Nerve growth factor, brain derived neurotrophic factor  [ **BDNF** ]   **BDNF**  and neurotrophin 3  [ **NTF3** ]  NT 3  [ **NTF3** ]  mRNAs were revealed in OLGs in vivo and in culture.  Ref: 12843289 J Neurosci, 2003
- BY MEANS OF IMMUNOCHEMISTRY AND IMMUNOHISTOCHEMISTRY, WE INVESTIGATED IN THE GUT OF TELEOSTEAN SPECIES THE PRESENCE AND LOCALIZATION OF THREE NEUROTROPHINS nerve growth factor NGF, brain derived neurotrophic factor  [ **BDNF** ]   **BDNF**  and neurotrophin NT 3  [ **NTF3** ] .  Ref: 12809982 Neurosci Lett, 2003
- In contrast, the expression levels of  **BDNF** , NT 3  [ **NTF3** ] , trkB and trkC were significantly higher at the rostral and caudal sites immediately adjacent to the maximal compression site.  Ref: 12774239 Acta Neuropathol Berl , 2003
- Prior exposure to MeHg had no effect on NT 3  [ **NTF3** ]  or  **BDNF**  stimulated PI hydrolysis in the cerebellum.  Ref: 12835095 Neurotox Res, 2000
- Cultured cortical progenitors express  **BDNF**  and NT 3  [ **NTF3** ] , as well as their receptors TrkB tyrosine kinase receptor B and TrkC.  Ref: 12832539 J Neurosci, 2003
- Here, we investigated the role of two endogenously produced growth factors, the neurotrophins brain derived neurotrophic factor  [ **BDNF** ]  and neurotrophin 3  [ **NTF3** ]  NT 3  [ **NTF3** ] , in the early progenitor to neuron transition.  Ref: 12832539 J Neurosci, 2003
- Moreover, trophic effects of OLG conditioned medium were partially blocked by K252a, a trk tyrosine kinase inhibitor, and by neutralizing anti  **BDNF**  or anti NT 3  [ **NTF3** ]  antisera, indicating that neurotrophins may mediate these effects, perhaps in concert with other signals.  Ref: 12843289 J Neurosci, 2003
- OBJECTIVES The aim of this study was to assess whether eosinophils of allergic and non allergic donors produce, store and release the neurotrophic factors NGF,  **BDNF**  and NT 3  [ **NTF3** ] .  Ref: 12752594 Clin Exp Allergy, 2003
- In this work the expression and anatomical localization of NGF,  **BDNF**  and NT 3  [ **NTF3** ]  and of corresponding high affinity receptors TrkA, TrkB full length and truncated TR isoforms, TrkC and of the low affinity p75 receptor were assessed in surgical samples from adult human lung by reverse transcriptase polymerase chain reaction RT PCR, Western blot and immunohistochemistry.  Ref: 12791675 Am J Respir Cell Mol Biol< MedlineTA>Am J Respir Cell Mol Biol, 2003
- In the retina, superior colliculus SC, primary visual cortex V1, hippocampus HIPP and cerebellum CBL, using a ribonuclease protection assay RPA, we examined expression of the mRNAs for nerve growth factor NGF,  **BDNF** , NT3  [ **NTF3** ] , NT4, ciliary neurotrophic factor CNTF and glial cell line derived neurotrophic factor GDNF.  Ref: 12855194 Brain Res Dev Brain Res, 2003
- Neurons secreted the brain derived neurotrophic factor  [ **BDNF** ]  and neurotrophin 3  [ **NTF3** ]  to induce neuron differentiation, while astrocytes secreted ciliary neurotrophic factor for astrocyte differentiation.  Ref: 12837625 Mol Cell Neurosci, 2003
- Immunoreactivities for  **BDNF** , NT 3  [ **NTF3** ] , trkB and trkC were preferentially localized in the gray matter, particularly in the anterior horn cells.  Ref: 12774239 Acta Neuropathol Berl , 2003
- Western blot analysis confirmed these results, showing that the lacrimal gland expresses NGF,  **BDNF** , NT3  [ **NTF3** ] , and NT4 as well as the NTrs TrkA, TrkB, and TrkC and the p75 protein.  Ref: 12882781 Invest Ophthalmol Vis Sci, 2003
- Acute exposure to 10 micro M MeHg increased basal PI hydrolysis in cortical slices and increased NT 3  [ **NTF3** ]  and  **BDNF**  stimulated PI hydrolysis in slices from the cerebellum.  Ref: 12835095 Neurotox Res, 2000
- RT PCR showed positive bands at the appropriate sizes for NGF,  **BDNF** , NT3  [ **NTF3** ] , and NT4, and for the receptors TrkA, TrkB, TrkC, and p75.  Ref: 12882781 Invest Ophthalmol Vis Sci, 2003
- The production, storage and release of the neurotrophins nerve growth factor, brain derived neurotrophic factor  [ **BDNF** ]  and neurotrophin 3  [ **NTF3** ]  by human peripheral eosinophils in allergics and non allergics.  Ref: 12752594 Clin Exp Allergy, 2003
- CONCLUSIONS Eosinophils store, produce and release NGF,  **BDNF**  and NT 3  [ **NTF3** ] .  Ref: 12752594 Clin Exp Allergy, 2003
- Neurotrophin 3  [ **NTF3** ]  NT 3  [ **NTF3** ]  and brain derived neurotrophic factor  [ **BDNF** ]   **BDNF**  stimulated PI hydrolysis in neocortical and cerebellar slices from neonatal rats in a concentration dependent manner 30 1000 ng ml.  Ref: 12835095 Neurotox Res, 2000
- Messenger RNAs encoding other neurotrophins, including nerve growth factor NGF and neurotrophin 3  [ **NTF3** ]  NT 3  [ **NTF3** ]  and the  **BDNF**  receptor trkB, were not selectively localized in neonatal VMN.  Ref: 12824057 Brain Res Mol Brain Res, 2003
